# Dysregulation of 2-oxoglutarate-dependent dioxygenases by hyperglycaemia: does this link diabetes and vascular disease?

**DOI:** 10.1186/s13148-020-00848-y

**Published:** 2020-04-28

**Authors:** Hannah L. H. Green, Alison C. Brewer

**Affiliations:** School of Cardiovascular Medicine & Sciences, King’s College London British Heart Foundation Centre of Research Excellence, London, UK

**Keywords:** Diabetes, Hyperglycaemia, 2-oxoglutarate-dependent dioxygenase, TET proteins, JmjC proteins, Oxygen, Cellular redox, Metabolism, DNA methylation, Histone methylation

## Abstract

The clinical, social and economic burden of cardiovascular disease (CVD) associated with diabetes underscores an urgency for understanding the disease aetiology. Evidence suggests that the hyperglycaemia associated with diabetes is, of itself, causal in the development of endothelial dysfunction (ED) which is recognised to be the critical determinant in the development of CVD. It is further recognised that epigenetic modifications associated with changes in gene expression are causal in both the initiation of ED and the progression to CVD. Understanding whether and how hyperglycaemia induces epigenetic modifications therefore seems crucial in the development of preventative treatments. A mechanistic link between energy metabolism and epigenetic regulation is increasingly becoming explored as key energy metabolites typically serve as substrates or co-factors for epigenetic modifying enzymes. Intriguing examples are the ten-eleven translocation and Jumonji C proteins which facilitate the demethylation of DNA and histones respectively. These are members of the 2-oxoglutarate-dependent dioxygenase superfamily which require the tricarboxylic acid metabolite, α-ketoglutarate and molecular oxygen (O_2_) as substrates and Fe (II) as a co-factor. An understanding of precisely how the biochemical effects of high glucose exposure impact upon cellular metabolism, O_2_ availability and cellular redox in endothelial cells (ECs) may therefore elucidate (in part) the mechanistic link between hyperglycaemia and epigenetic modifications causal in ED and CVD. It would also provide significant proof of concept that dysregulation of the epigenetic landscape may be causal rather than consequential in the development of pathology.

## Introduction

A startling increase in obesity driven by a sedentary lifestyle and high-calorie diet has resulted in a worldwide epidemic of type 2 diabetes (T2D) that is set to further rise dramatically [1, 2]. Thus, current estimates suggest that by 2045, 700 million people will be living with diabetes [2]. The hyperglycaemia characteristic of diabetes represents a major risk factor for the development of cardiovascular disease (CVD) which is ultimately the most common cause of mortality in affected individuals [3]. Further, the phenomena of hyperglycaemic memory, where phenotypic alterations persist despite the restoration of normal glycaemic control, renders CVD a life-long risk to individuals who have been affected by diabetes. This predisposition to CVD additionally affects the offspring of mothers who exhibit gestational diabetes (defined as glucose intolerance resulting in hyperglycaemia with onset or first discovery in pregnancy), cases of which have similarly burgeoned with the obesity crisis [4]. Such developmental priming has also been shown to exhibit inheritance to the second (F2) generation and therefore additionally compounds the current problems onto the future population [5, 6]. The economic burden associated with this is vast and in the current economic climate, unaffordable. It is therefore vital that better preventative and therapeutic clinical strategies are developed. The underlying mechanisms which predispose individuals who are, or have in the past been exposed to, hyperglycaemia must therefore be determined.

### Hyperglycaemia, endothelial dysfunction and cardiovascular disease

Diabetes is associated with the development of complications affecting both the microvasculature (diabetic retinopathy, neuropathy and nephropathy) and macrovasculature (peripheral artery disease, cardiomyopathy, myocardial infarction and stroke) [7]. Macrovascular complications are primarily responsible for the reduced life expectancy of diabetic patients; T2D is associated with a two to sixfold greater risk of cardiovascular mortality than amongst non-diabetics [8, 9] and similarly high relative risks are reported for type 1 diabetics [10]. It is well established that the dysfunction of the vascular endothelium, which comprises the one cell thick, innermost layer of the vascular wall is both a hallmark of vascular disease and is critical in the development of both macrovascular and microvascular pathologies [11, 12]. Endothelial dysfunction (ED) is characterised by a proinflammatory, prothrombotic state with impaired vasodilation [11, 13, 14] and a reduced bioavailability of the critical vascular mediator, nitric oxide (NO) [11]. Given the direct contact between endothelial cells (ECs) and blood, it is not surprising that they are critically affected by metabolic changes in blood plasma such as hyperglycaemia. Indeed, a significant body of evidence from in vitro studies suggests that hyperglycaemia per se is causative in the development of ED [15–27]. Thus, it has been shown that even short-term exposure of ECs to high glucose is sufficient to induce monocyte adhesion [15], promote endothelial to mesenchymal transition [16], activate prothrombotic signalling [20], reduce eNOS activity [21] and increase apoptosis [22]. High-glucose culture conditions also enhanced the response of human umbilical vein endothelial cells (HUVECs) to the pro-inflammatory stimulus interleukin-1β, resulting in augmented endothelial ICAM-1 and VCAM-1 expression and promoting increased leukocyte adhesion [26], further demonstrating the effect of hyperglycaemia upon the inflammatory responses characteristic of ED.

### Endothelial dysfunction and epigenetics

The mechanisms whereby chronic hyperglycaemia mediate ED have classically been attributed to involve the overproduction of mitochondrial-generated superoxide via PKG activation and upregulation of the hexosamine and polyol pathways leading to the production of advanced glycosylation end-products (AGEs) [28–30]. However, these mechanisms cannot account for the observation of hyperglycaemic memory, including developmental priming. It is now evident from epidemiological and clinical studies that the development of T2D and associated CVD is correlated with, and in part depends upon, environmentally influenced epigenetic changes which modulate gene expression (reviewed in [31]). The term epigenetics has come to mean heritable alterations in gene expression and phenotype that do not involve changes in the primary DNA sequence [32]. Epigenetic mechanisms thus involve stable cellular modifications, capable of governing functional changes in gene expression patterns in response to external environmental stimuli, or as part of normal development [33]. Epigenetic modifications include changes to histone post-translational modifications, non-coding RNA and DNA methylation patterns, which serve to activate or repress transcription of a gene [32]. In essence, epigenetic modifications govern remodelling of chromatin, placing it in a conformation which is ‘open’ (euchromatin) or ‘closed’ (heterochromatin) to transcription factors and other transcription machinery [32]. The epigenetic landscape of a cell is thus a major regulator of gene transcription and accounts for the vastly different phenotypes and functions of cells containing identical DNA, providing a link between phenotype and genotype.

### Hyperglycaemia and epigenetic regulation

Although not documented here, the (ever-increasing) recognised associations between changes in the epigenome and T2D-related CVD are the subject of many excellent reviews [34–37]. Further, the study of the regulation of these epigenetic changes in the aetiology and progression of ED and CVD in diabetes is an emerging field. The influence of high glucose upon the EC epigenetic landscape and transcriptome has clearly been demonstrated in vitro. Thus in an important study, it was shown that the incubation of human aortic endothelial cells (HAECs) in high glucose (30 mM) for 2 days resulted in significant changes in the expression of genes associated with diabetes and vascular complications, many of which correlated with (histone and DNA) epigenetic changes at these loci [38]. Thus, hyperglycaemia per se can mediate epigenetic changes that associate with changes in transcription in ECs.

Of particular significance in this regard is the emerging intimate relationship between metabolic alterations and epigenetic modifications in both health and disease [39, 40]. The epigenome must be maintained by the balance of the actions of a variety of epigenetic modifiers, including the ‘writers’ and ‘erasures’ of epigenetic marks such as posttranslational histone modifications and DNA methylation. Crucially, many of these modifiers use key metabolites (including S-adenosyl methionine (SAM), ATP and acetylCoA) as obligate substrates or co-factors, suggesting a mechanistic link between metabolic changes and epigenetic (dys)regulation of gene transcription (reviewed in [40]). It is therefore tempting to speculate that metabolic changes induced by the hyperglycaemia characteristic of diabetes may affect the activities of these epigenetic modifiers by altering the availability of their substrates (and/or co-factors). In this regard, it is particularly intriguing that the enzymes which facilitate the demethylation of both DNA and histones are members of the superfamily of 2-oxoglutarate-dependent dioxygenases (2-OGDDs). These require the tricarboxylic acid (TCA) metabolite, α-ketoglutarate (2-OG) and molecular oxygen (O_2_) as substrates and Fe (II) as a co-factor.

### DNA methylation

DNA methylation represents the best characterised epigenetic mark to date. It occurs via the transfer of a methyl group from the universal methyl donor; SAM by a DNA methyltransferase (DNMT) to the fifth position of a cytosine ring to form 5-methylcytosine (5mC) [41]. This modification predominantly occurs at CpG sites, which are often found clustered in ‘CpG islands’ of regulatory elements such as promoters and enhancers [41]. The pattern of DNA methylation is regulated by writers and erasers which add and remove methyl groups respectively. Thus, members of the DNMT family act as writers; DNMT3A/DNMT3B are involved in de novo methylation, whilst DNMT1 is involved in the maintenance of the methyl mark in subsequent mitotic divisions [42, 43]. DNMTs are essential for development [42, 43] and play an important role in maintaining genomic integrity [44]. DNA methylation is ‘read’ by various chromatin-binding proteins containing methyl-CpG-binding domains which can form repressive complexes to silence transcription [41].

**Fig. 1:**
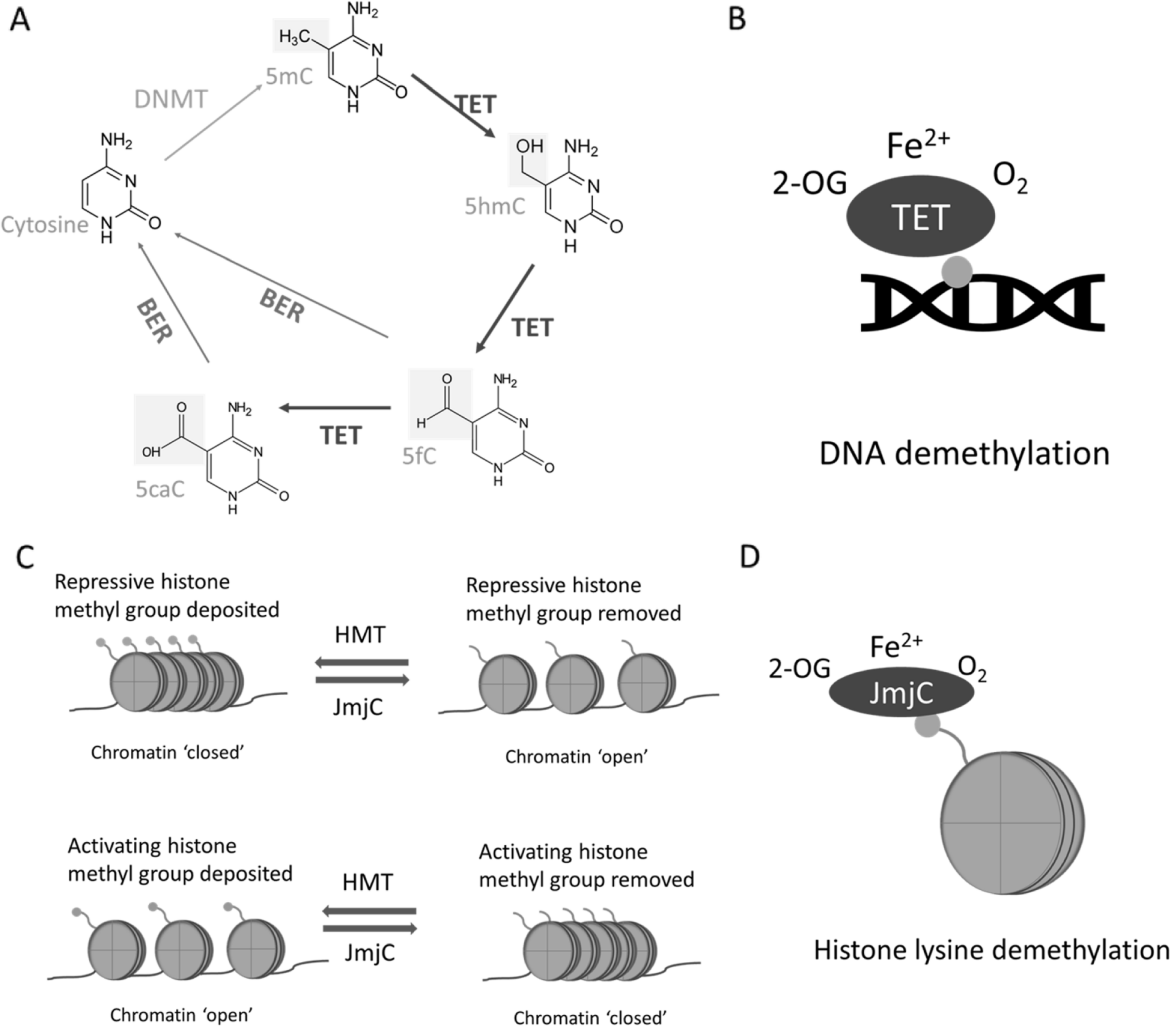
Action of ten-eleven translocation (TET) and Jumonji C (JmjC) enzymes. **a** Cytosine is converted to 5-methylcytosine (5mC) by the addition of a methyl group by DNA methyltransferase enzymes (DNMTs). TET enzymes successively oxidise 5mC to 5-hydroxymethylcytosine (5hmC), 5-formylcytosine (5fC) and 5-carboxylcytosine (5caC). Base excision repair (BER) machinery excise and replace the intermediates with unmethylated cytosine. **b** TET enzymes require 2-oxoglutarate (2-OG), Fe^2+^ and molecular oxygen (O_2_) for their DNA demethylase activity. **c** Histone methyltransferases (HMTs) can deposit either repressive or activating methyl marks depending on which site is methylated and to what extent (mono/di/trimethylation). This results in a ‘closed’ and ‘open’ conformation of chromatin, respectively. Hence, the removal of methyl groups by JmjCs can result in the activation or repression of a gene. **d** JmjC enzymes require 2-oxoglutarate (2-OG), Fe^2+^ and molecular oxygen (O_2_) for their histone lysine demethylase activity

Traditionally, DNA methylation was believed to be a permanent epigenetic modification which could only be lost passively during DNA replication [45]. However, relatively recently, this notion was challenged by the discovery of ten-eleven translocation (TET) enzymes: TET1, TET2 and TET3 [46] which act as erasers of this modification by active DNA demethylation mechanisms [47, 48]. TETs catalyse the successive oxidations of 5mC to 5-hydroxymethylcytosine (5hmC), 5-formylcytosine (5fC) and 5-carboxylcytosine (5caC) (Fig. 1a) [47, 48]. These variants can be either lost passively during DNA replication or actively removed and replaced with unmethylated cytosine by base excision repair machinery including thymine DNA glycosylase (TDG) (Fig. 1a) [48]. Not only is the removal of 5mC important for reversing transcriptional repression, but it is now recognised that the oxidised intermediates formed by TETs may perform distinct regulatory functions in their own right [49]. Evidence for this comes from differential genomic positions of these oxidised derivatives [50, 51], the existence of distinct proteins which recognise and read the cytosine variants [52], and reported roles for 5fC and 5caC in fine-tuning the rate of transcription by RNA polymerase II [53]. As stated above, these TET proteins are 2-OGDDs which require 2-OG and molecular oxygen (O_2_) as substrates and Fe (II) as a co-factor (Fig. 1b).

### Histone methylation

In chromatin, DNA is wrapped around histone proteins to form nucleosomes, which in turn form a ‘beads on a string’ structure that folds to form a 30 nm fibre [54]. Further condensation results in 250 nm supercoiled fibres which make up the chromatid of a chromosome [54]. The canonical nucleosome contains a tetramer of histone H3 and H4, interacting with two dimers of histone H2A-H2B [54]. The tails of these histones protrude from the nucleosome and can be post-translationally modified by methylation, acetylation, phosphorylation, ubiquitination, SUMOylation or O-GlcNAcylation to alter the interaction of DNA with the nucleosome to facilitate or repress gene transcription [55]. Of these, the most well studied are histone methylation and acetylation [41]. Histone acetyltransferases (HATs) and histone deacetylases (HDACs) respectively comprise the writers and erasers of the histone acetylation mark, which occurs predominantly at lysine residues [56]. This neutralises the positive charge of lysine, causing disruption of electrostatic interactions between the histone and DNA, typically resulting in enhanced transcription [57]. The readers of histone acetylation are chromatin-binding proteins containing an acetyl-lysine reader domain, which participate in transcriptional activation [41].

Histone methylation at lysine or arginine residues is conferred by the transfer of a methyl group (again donated from SAM) by histone methyltransferases (HMTs) and (as in the case of DNA methylation) can be actively erased by histone demethylases (Fig. 1c) [56]. Compared to histone acetylation, histone methylation patterns are more complex and have more diverse effects depending on the position and extent of methylation [57]. Thus there are three degrees (mono-, di- and tri-) of methylation which occur on lysine residues, while arginine residues can be monomethylated and symmetrically or asymmetrically demethylated [58]. Further, methylation at specific amino acid residues within the histone tails recruit either activating or repressive complexes. For example, trimethylation of histone 3 lysine 4 (H3K4_me3_) is generally associated with active transcription (euchromatin), whilst trimethylation of lysine 9 or 27 (H3K9_me3_, H3K27_me3_) are associated with transcriptionally repressive state (constitutive and facultative heterochromatin respectively, Fig. 1c) [57]. The complexity of the histone methyl marks is reflected by the large number of HMTs and demethylases which observe a high degree of specificity for the specific lysine/arginine and degree of methylation being modified (reviewed in [59]). In the case of histone demethylases, there are two evolutionarily conserved families which employ distinct mechanisms, both of which involve oxidation of the methyl group. Lys-specific demethylases (LSDs) catalyse a flavin adenine dinucleotide (FAD)-dependent oxidation reaction [60]. By contrast, the Jumonji C demethylases (JmjCs) are, like the TET enzymes, members of the 2-OGDD superfamily which employ molecular oxygen and 2-OG as substrates (Fig. 1d) [61].

## Hyperglycaemia and the regulation of TETs and JmjCs

The dependency of the epigenetic demethylases; TETs and JmjCs, upon O_2_, 2-OG and Fe^2+^ is particularly intriguing in the context of diabetes, as the availability of each of these factors is potentially affected by hyperglycaemia. We, here, review the current evidence of dysregulation of these epigenetic erasers by hyperglycaemia in the endothelium and explore the known biochemical mechanisms which might underlie this dysregulation.

### Hyperglycaemia and availability of molecular oxygen

As stated above, molecular oxygen is an obligate substrate for all 2-OGDDs, of which the best characterised are the related hypoxia-inducible factor-1prolyl hydroxylases (HIF-PHD1–3). Under normoxic conditions, PHDs hydroxylate HIF1α, leading to its association with von Hippel-Lindau and subsequent degradation [62]. The inhibition of PHDs by relative hypoxia, due to their requirement for molecular oxygen, leads to HIF stabilisation and HIF-dependent activation of transcription [62]. Studies performed to determine the *K*_M_ values for O_2_ of these enzymes suggest that their activities may be modulated by fluctuations in [O_2_] over physiological ranges, at least in some tissues, further substantiating their roles as bona fide O_2_ sensors [63].

Physiological normoxia within eukaryotic cells is maintained by the balance of its diffusional uptake and metabolic usage, of which oxidative phosphorylation (OxPhos) represents by far the largest consumer [64]. An upset in this metabolic flux will therefore impact upon the intracellular [O_2_] and potentially modulate the activities of enzymes which use O_2_ as a substrate, including the TET and JmjC demethylases. Despite being positioned in a typically highly oxygenated environment, the primary method of adenosine triphosphate (ATP) production in ECs is glycolysis, which is estimated to generate 85% of endothelial ATP [65, 66]. Thus, compared to other cell types, ECs are much less reliant on oxidative processes in the mitochondria [66] which account for less than 5% of the total cell volume (compared to, for instance, approximately 25% of the hepatocyte cell volume) [64]. Instead, glycolysis allows rapid and sufficient energy production for the supply of homeostatic processes even under hypoxic conditions, such as proliferation and migration required for angiogenesis [65].

**Fig. 2:**
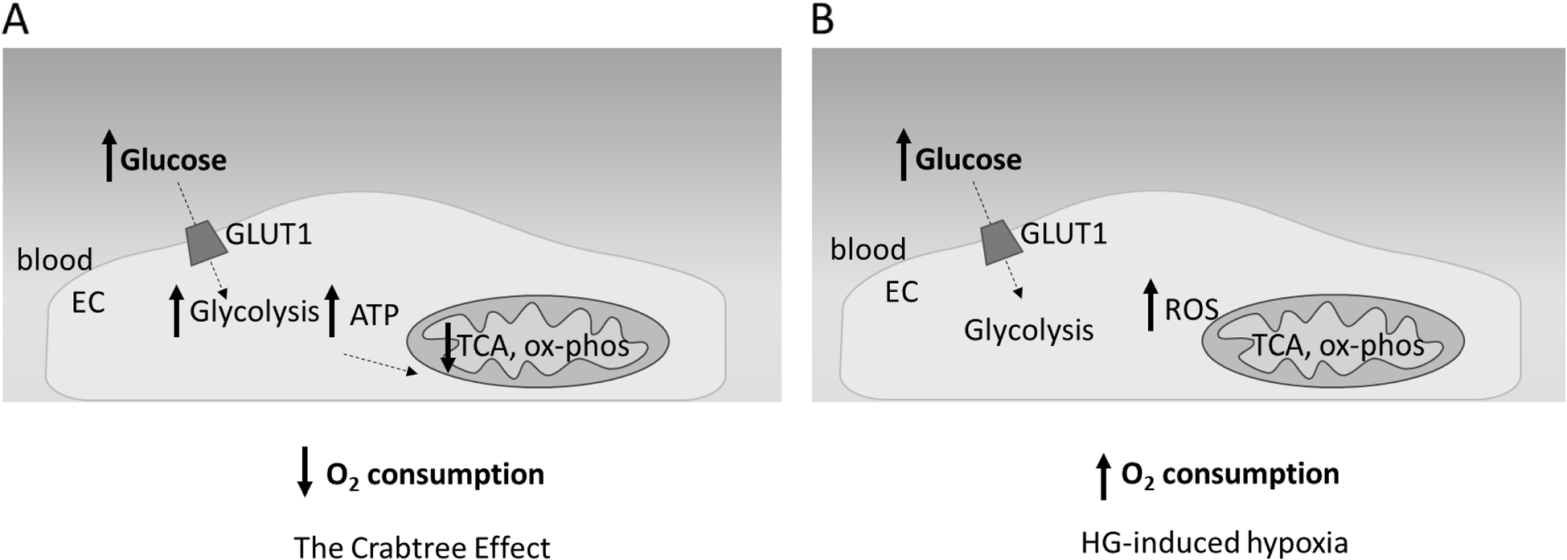
Potential effects of hyperglycaemia on endothelial oxygen consumption. **a** Endothelial cells (EC) have been reported to decrease oxygen consumption in hyperglycaemia (HG) (the Crabtree Effect), perhaps due to ATP requirements being met through glycolysis, resulting in a decreased requirement for ATP production by the tricarboxylic acid cycle (TCA) and oxidative phosphorylation (ox-phos). **b** HG has also been reported to increase oxygen consumption and cause intracellular hypoxia perhaps in a reactive oxygen species (ROS)-dependent manner

To maintain physiological normoxia, the relative consumption of O_2_ through OxPhos (and glycolysis) is tightly regulated in response to different levels of oxygenation [67] and this balance can be dysregulated in a cell-specific manner under hyperglycaemic conditions. The metabolic response of ECs to increased glucose abundance is currently not fully defined. Glucose enters ECs by facilitated diffusion via GLUT 1[68] in a manner that is not regulated by insulin [69] and therefore intracellular levels increase in hyperglycaemia (Fig. 2). Glucose utilisation is controlled by its flux through glycolysis, the TCA cycle, OxPhos, and alternative metabolic pathways [69]. Under hyperglycaemic conditions, ECs have been shown to exhibit the ‘Crabtree effect’, where the O_2_ consumption rate of cells is *decreased* despite high glucose concentrations, potentially resulting in higher intracellular [O_2_] (Fig. 2a) [70]. This is thought to be due to a higher glycolytic flux resulting in significant ATP production and increased substrate-level phosphorylation, which in turn acts to decrease the requirement for ATP produced by OxPhos, thus reducing O_2_ consumption (Fig. 2a) [70]. An increase in anaerobic glucose metabolism is also evidenced by reports of elevated lactate production and resultant acidosis in ECs cultured under high glucose conditions and in peripheral tissues of diabetic patients [31, 70–72].

By contrast, endothelial O_2_ consumption rate has, in some reports, also been reported to *increase* upon exposure to high glucose culture conditions (Fig. 2b) [73]. Using pimonidazole staining as an indicator of hypoxia, Sada et al. showed significantly elevated hypoxic staining in bovine aortic endothelial cells (BAECs) cultured in media containing 25 mM glucose compared to that containing 5 mM glucose [73]. This effect was diminished by blocking the mitochondrial electron transport chain or overexpressing manganese superoxide dismutase (SOD), indicating a potential involvement of mitochondrial ROS in this finding [73]. This study also reported intensified hypoxic staining in the glomeruli of diabetic mice, in vivo. Increased O_2_ consumption under hyperglycaemia leading to activation of HIF signalling and hypoxia-induced pathways was also demonstrated in isolated rat islets and pancreatic beta cells in vitro [74] and it is thought that this may contribute to apoptosis and oxidative stress-associated cellular damage in diabetic patients [75]. Furthermore, the pathological effects of hyperglycaemia and hypoxia in diabetes are thought to be additive. Thus, in retinas isolated from normal rats, high glucose and hypoxia were shown to increase cytosolic free NADH via distinct metabolic mechanisms to potentially fuel biochemical pathways associated with the progression of diabetic retinopathy [76]. Hyperglycaemia can also worsen tissue responses to hypoxia. For example, Capla et al. observed decreased mobilisation of endothelial progenitor cells from the bone marrow of diabetic mice following ischaemic injury, and demonstrated in vitro that these cells displayed impaired adhesion, migration and proliferation in response to hypoxic culture conditions [77].

The altered cellular availability of O_2_ in hyperglycaemia, whether increased or decreased, could, as stated above, potentially alter the activities of TETs and JmjCs in ECs, dependent upon the *K*_M_s of the proteins for O_2_ and the (patho)physiological ranges of O_2_ to which they are exposed. The physiologically normoxic intracellular environment of ECs has been determined to be 3.5-4 kPa [64]. Although not yet comprehensively completed, some studies have been performed to determine the *K*_M_s of individual purified TET and JmjC proteins which suggest that, (at least) in some cases, their activities may be modulated by moderate fluctuations in physiological levels of O_2_ [78, 79]. There is also increasing evidence from cellular studies that relative hypoxia can act to inhibit the activities of both TETs and JmjCs [80–83]. Further studies need to be carried out to determine whether the activities of these demethylases may be modulated by any changes in consumption and therefore availability of O_2_ resulting from hyperglycaemia in ECs.

It is also noteworthy that, perhaps paradoxically, the transcription of TET1, together with that of some JmjC demethylase family members; JMJD1A, JMJD2B and JARID1B, is under the positive control of HIF or hypoxia-driven pathways [84–87]. Thus, a reduction in intracellular [O_2_] may result in a decrease in enzymatic activity that is balanced by a (potentially later-onset) increase in expression, suggesting a complex mode of hypoxia-induced regulation.

### Hyperglycaemia and metabolism

**Fig. 3:**
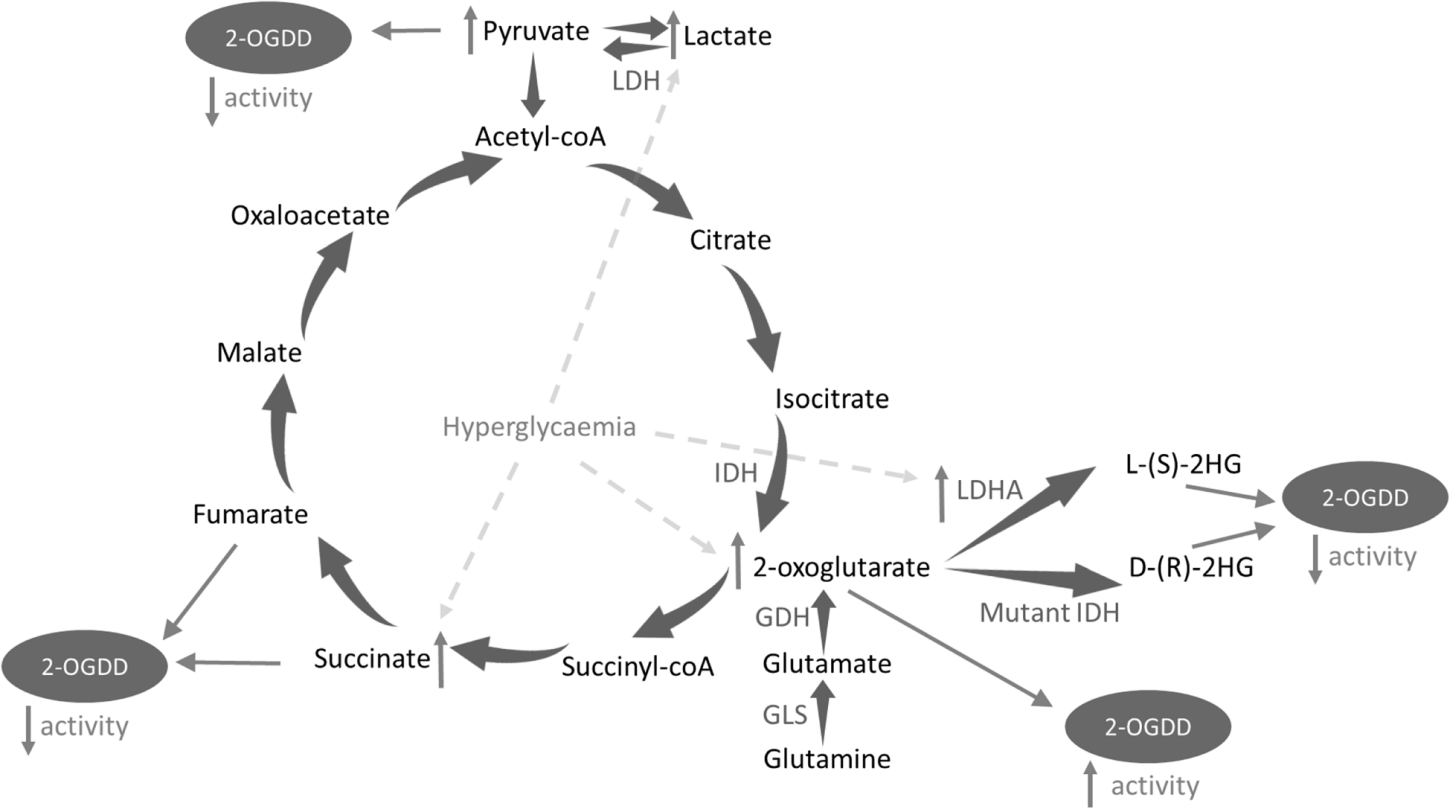
Dysregulation of metabolites by hyperglycaemia. Hyperglycaemia has been reported to increase levels of lactate and succinate, which have been reported to decrease 2-oxoglutarate dependent dioxygenase (2-OGDD) activity. Increased flux through the tricarboxylic acid cycle may occur during hyperglycaemia, which could alter intracellular levels of 2-oxoglutarate, fumarate and succinate. High levels of 2-hydroxyglutarate (2HG), formed by the action of lactate dehydrogenase (LDH)-A on glutamine-derived 2-OG, or by mutant isocitrate dehydrogenase (IDH), is also reported to inhibit 2-OGDD activity. Abbreviations: glutamate dehydrogenase (GDH), glutaminase (GLS)

The dependence of 2-OGDDs, including TETs and JmjC demethylases on their co-substrate, 2-OG, positions them at the interface between cellular metabolism and epigenetic regulation. It may therefore be speculated that they could be involved in mediating changes in gene expression in response to an altered metabolic state induced by hyperglycaemia. Perhaps significantly in this regard, injection of mice with glucose, glutamine or glutamate rapidly increased TET activity, as assessed by an increase in hepatic 5hmC levels, concomitant with elevated hepatic 2-OG levels (Fig. 3) [88]. Similar increases in 5hmC were observed in kidneys and skeletal muscle [88]. Both TETs and JmjCs have conversely been shown to be strongly inhibited via steric competition of 2-OG by the downstream TCA metabolites, succinate and fumarate (Fig. 3) [89, 90]. Accordingly, accumulation of fumarate and succinate following knockdown of fumarate hydratase and succinate dehydrogenase enzymes in HEK293T cells and mouse liver resulted in altered DNA and histone methylation patterns consistent with altered TET and JmjC demethylase activity (Fig. 3) [89]. Changes in the flux through the TCA cycle resulting in altered availability of these metabolites could therefore have profound effects on these epigenetic modulators and hence on cell function. Indeed, dysregulation of the TCA cycle has been reported to be an early manifestation of endothelial dysfunction [91].

Although ATP synthesis primarily occurs via glycolysis in ECs, the mitochondria remain active, performing vital roles in NO generation, maintaining calcium homeostasis and regulating ROS production, in addition to some ATP generation [92]. Additionally, as stated above, metabolites generated by the TCA cycle act as cofactors or inhibitors of many enzymes including epigenetic modifiers [89, 90]. Reports of the effects of hyperglycaemia upon O_2_ consumption, and hence flux through the TCA cycle in high glucose conditions are, as stated above, varied [70, 73]. Further, to our knowledge, studies of 2-OG, succinate and fumarate levels in response to high glucose have not been conducted in ECs. However, the culture of isolated rat pancreatic islets in high glucose conditions was demonstrated to increase islet succinate content by 40% [93]. Given their potential effects upon the epigenetic landscape, the effects of hyperglycaemia upon TCA flux and steady-state metabolite levels in ECs warrants further investigation.

Another intermediate metabolite which has been shown to function as a competitive inhibitor of 2-OGDDs including TETs and JmjCs is 2-hydroxyglutarate (2HG) [94]. This is a chiral molecule which exists as a D-or L-enantiomer, both of which have been shown to inhibit 2-OGDDs (Fig. 3) [94–97]. Normal cells generate small quantities of both enantiomers (via poorly-understood pathways), but intracellular levels are typically kept low by the actions of D- and L-2-hydroxyglutarate dehydrogenases [98]. Intriguingly, D-2HG, produced by mutant gain-of-function isocitrate dehydrogenase is considered an ‘oncometabolite’ [99]. High levels of D-2HG are thus associated with and contribute to cancer pathogenesis, via inhibition of 2-OGDDs, including JmjC and TET enzymes [100]. More recently, it has been shown that L-2HG is selectively generated by cells under hypoxia and that this increase is both necessary and sufficient for increased histone methylation marks, acting through inhibition of specific JmjC proteins [100]. Biochemically, the hypoxia-induced L-2HG was found to be generated from glutamine-derived 2-OG, via the action of lactate dehydrogenase (LDH)-A, demonstrating promiscuous substrate usage. It is noteworthy that the levels of LDH have been shown to increase in diabetic rabbits, and that mammalian LDH enzymes, other than LDH-A, are known to have the potential to produce L-2HG under more acidic environments [101], such as those resulting from the increased lactic acid production in hyperglycaemia (Fig. 3) [102]. In another report, levels of L-2HG were similarly shown to accumulate in response to hypoxia, via the action of malate dehydrogenase (MDH) on (increased levels of) mitochondrial 2-OG [103] resulting from TCA dysfunction and mitochondrial stress. In turn, the increased levels of L-2HG acted to inhibit electron transport and glycolysis. The relationship between hyperglycaemia, hypoxia, altered TCA flux and the regulation of epigenetic modifiers by L-2HG is intriguing and remains to be explored.

The observed increases in endothelial lactate production resulting from increased glycolytic flux during hyperglycaemia [70–72] also has the potential to influence 2-OGDD activity. Thus, it has been reported that that the uptake of high levels of exogenous lactate increased HIF-1α expression in ECs, leading to angiogenesis, independently of hypoxia [104]. Mechanistically, it was proposed that the conversion of lactate to pyruvate by LDH-B leads to competitive substrate inhibition of PHD2 by pyruvate, reducing the ability of PHD2 to trigger HIF-1α degradation [104]. Investigation of the regulation of other 2-OGDDs by glycolysis metabolites has not yet been conducted. However, this provides further support to the idea that 2-OGDDs may act as sensors of the metabolic status of ECs.

### Hyperglycaemia, cellular redox and iron homeostasis

2-OGDDs require Fe^2+^ as a cofactor for their catalytic activity, so the bioavailability of iron in its reduced form is important in maintaining their function [105, 106]. In evidence of this, two groups identified that supplementation with ascorbic acid (vitamin C), which promotes the reduction of Fe^3+^ to Fe^2+^, enhanced TET-mediated oxidation of 5mC [105, 106]. The histone demethylation activity of JmjCs has similarly been demonstrated to increase with vitamin C supplementation [107–109].

**Fig. 4:**
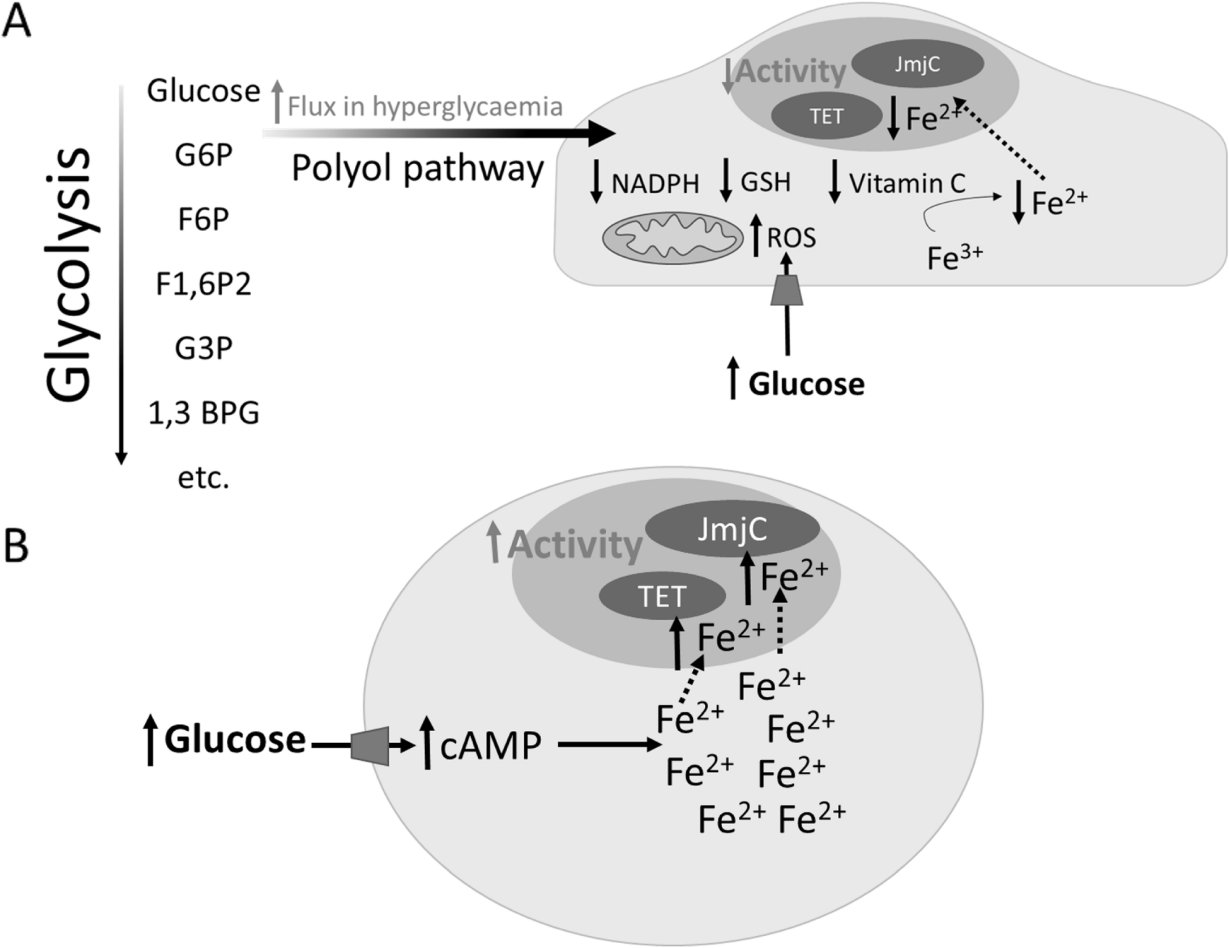
Hyperglycaemia may affect ten-eleven translocation (TET) and Jumonji C (JmjC) activity by altering Fe^2+^ availability. **a** Hyperglycaemia increases flux through the polyol pathway, which consumes reduced nicotinamide adenine dinucleotide phosphate (NADPH). Reduced glutathione (GSH) and vitamin C levels may decrease as a result, decreasing the regeneration of Fe^2+^ which is required by TET and JmjC enzymes. Increased generation of reactive oxygen species (ROS) in hyperglycaemia may also affect Fe^2+^ regeneration. **b** An increase in cyclic adenosine monophosphate (cAMP) levels in hyperglycaemia may increase the intracellular labile Fe^2+^ pool and thus increase TET and JmjC activity. Abbreviations: glucose-6-phosphate (G6P), fructose-6-phosphate (F6P), Fructose-1,6-bisphosphate (F1,6P2), glyceraldehyde-3-phosphate (G3P), 1,3-bisphosphoglycerate (1,3 BPG)

The bioavailability of Fe^2+^ in vivo is determined by multiple mechanisms which regulate cellular redox and iron homeostasis (transport, storage and metabolism) which may all impact upon the activities of TETs and JmjCs [110]. In hyperglycaemia, the cellular redox balance becomes perturbed, favouring excessive production of reactive oxygen species (ROS) and overloading the antioxidant defences which usually counteract this (Fig. 4a) [111]. A major source of hyperglycaemia-induced ROS production in ECs is the mitochondrial electron transport chain [111]. This is evidenced by the finding that the use of mitochondrial complex II inhibitors or oxidative phosphorylation uncouplers prevents endothelial ROS production [30, 112] ROS production in hyperglycaemia has also been reported to be mediated by increased NADPH oxidase activity [113, 114], eNOS uncoupling [115] and xanthine oxidase activity [116]. Hyperglycaemia-induced formation of AGEs induces an inflammatory response which can also generate ROS [117]. The direct effects of ROS-generating systems upon the activities of the epigenetic modifiers has so-far been little investigated. However, in one study, the administration of 150 μM H_2_O_2_ to epithelial cells resulted in increased global levels of several methylation marks (purported to be due to attenuated JmjC activity) together with reduced (global) TET activity [118]. By contrast, an oxidant-dependent *increase* in TET activity, in response to high glucose exposure, has been reported in bovine retinal endothelial cells [119]. Thus, a glucose-induced activation of TET2 was observed, as evidenced by higher 5hmC levels specifically at the matrix metalloproteinase-9 (MMP-9) gene promoter, which could be ablated by the addition of a mitochondrial SOD mimetic [119]. The significance of this (locus-specific) effect of hyperglycaemia-induced ROS in respect of the global levels of 5hmC and TET activity are at present unclear.

Steady-state cellular redox is often measured by ratios of oxidised and reduced forms of NAD and glutathione (NADH/NAD+ and GSH/GSSG respectively). The delicate balance of these agents determines the ability of a cell to undergo oxidation and reduction reactions which are crucial for many cellular processes including iron metabolism [120]. When ECs are exposed to hyperglycaemia, excess glucose is utilised by the polyol pathway, which consumes NADPH and generates NADH, resulting in an abnormal NADH/NAD+ balance [121]. Furthermore, this impairs generation of the reduced form of glutathione by glutathione reductase, which requires NADPH for its activity [121]. Glutathione is a major cellular redox buffer which, amongst many other functions, is responsible for regenerating the reduced form of vitamin C, necessary for optimal 2-OGDD activity (Fig. 4a) [122].

Interestingly, cyclic AMP (cAMP) has been shown to increase 5hmC levels by increasing the labile Fe^2+^ pool of primary Schwann cells, HEK293 and mouse embryonic fibroblasts [123]. Accordingly, TET activity is augmented by the increased availability of the Fe^2+^ cofactor, leading to increased detection of 5hmC [123]. Whether cAMP-dependent signalling acts to increase intracellular labile Fe^2+^ via increased iron uptake, storage, or conversion of Fe^3+^ to Fe^2+^, is not currently understood, but acidification of endosomes has been suggested to play a role [123]. Although changes in cAMP levels in hyperglycaemic conditions have not been quantified in ECs, high glucose exposure has been shown to increase cAMP levels in mesangial cells, which uptake glucose in a similar fashion to ECs*,* via GLUT-1 [124]. Therefore, hyperglycaemia-induced disturbances in cellular redox and iron homeostasis may be factors which potentially could mediate the activities of both TETs and JmjCs, due to their dependence on Fe^2+^ (Fig. 4b).

### Hyperglycaemia and non-catalytic roles of 2-OGDD demethylases

**Fig. 5:**
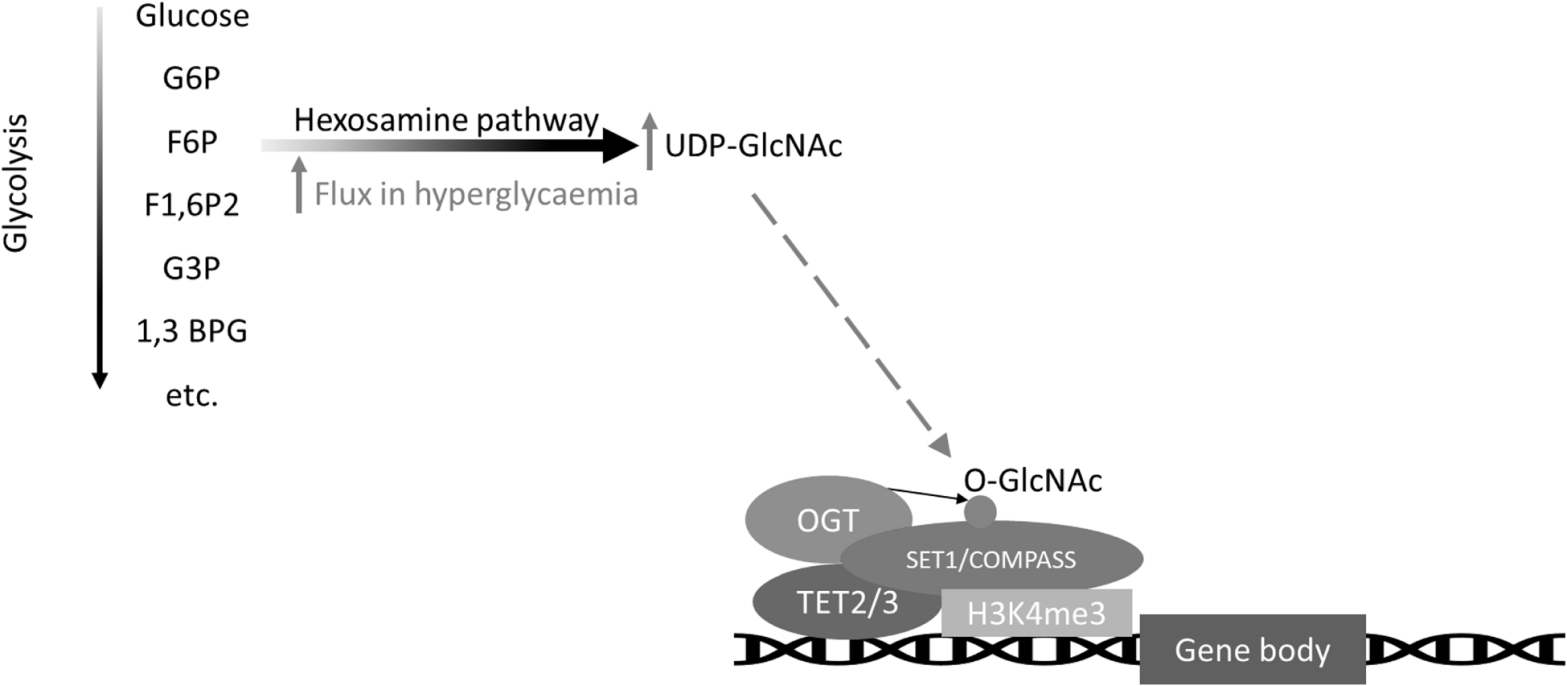
Hyperglycaemia may influence ten-eleven translocation (TET)-dependent O-GlcNAcylation of the histone methyltransferase SET1/COMPASS complex. TET2 and TET3 interact with O-GlcNAc Transferase (OGT), promoting its O-GlcNAcylation of the SET1/COMPASS complex which mediates trimethylation of histone H3 lysine 4 (H3K4me3). Hyperglycaemia increases hexosamine pathway flux so increases production of the substrate UDP-GlcNAc. Abbreviations: glucose-6-phosphate (G6P), fructose-6-phosphate (F6P), fructose-1,6-bisphosphate (F1,6P2), glyceraldehyde-3-phosphate (G3P), 1,3-bisphosphoglycerate (1,3 BPG)

There is an increasing body of research to suggest that TETs perform some epigenetic regulatory functions, which are distinct from their catalytic activity, by serving a scaffolding role via binding to DNA and recruiting other proteins or complexes [125–129]. These highlights the potential cross-talk between specific TET proteins and the regulation of histone methylation. Thus, physical and/or functional interactions between TET1 and TET2 and components of the Polycomb repressive complex 2 (PRC2), which mediates the methylation of H3K27 have been demonstrated [126, 130, 131]. Further, all 3 TET proteins have been shown to physically interact with O-GlcNAc Transferase (OGT) [127–129, 132]. A proven specific regulatory target of O-GlcNAcylation by (at least some) TET-OGT complexes is host cell factor 1 (HCF1), which is an integral element of the histone H3K4 methyltransferase SET1/COMPASS complex [127]. The substrate for both N- and O-linked glycosylation of proteins is UDP-GlcNAc, which is generated by the hexosamine biosynthesis pathway [133]. This is noteworthy with respect to hyperglycaemia because flux through the hexosamine pathway is increased by high glucose concentrations [133], likely resulting in higher UDP-GlcNAc production and subsequent GlcNAcylation of proteins (Fig. 5). Accordingly, O-GlcNac levels have been shown to be significantly increased in the aortae of obese, insulin-resistant mice compared to lean mice [134] and in rat aortic smooth muscle upon culture in high glucose media [135]. Thus, increased O-GlcNAcylation of SET1/COMPASS, resulting in elevated methylation of H3K4 may represent another mechanism whereby hyperglycaemia may influence epigenetic modifications, in a TET-dependent fashion.

### Transcriptional and post-translational regulation of TETs and JmjCs by hyperglycaemia

The activities and functions of TET and JmjC demethylases are not only regulated by the availability of their cofactors and substrates. Thus, like many proteins, their levels of activity within a cell are subject to both changes in their expression and post-translational modifications. Using a zebrafish model of diabetes, induced by streptozotocin injection, Dhliwayo et al. observed that hyperglycaemia induced a 5-10 fold increase in RNA expression of TET1, TET2 and TET3 in fin samples within 1 week, which persisted to 3 weeks after induction of hyperglycaemia [136]. This was supported by a significant global accumulation of 5hmC [136]. Administration of poly(ADP-ribose) polymerase (PARP) inhibitors rescued the hyperglycaemia-induced impairment of fin regeneration and prevented the increase in 5hmC, suggesting a role for PARPs in regulating TETs [136]. The authors suggest that this is due to suppression of TET expression, as PARP1 activity has previously been reported to be required for TET1 transcriptional upregulation in developing mouse primordial germ cells [137].

**Fig. 6:**
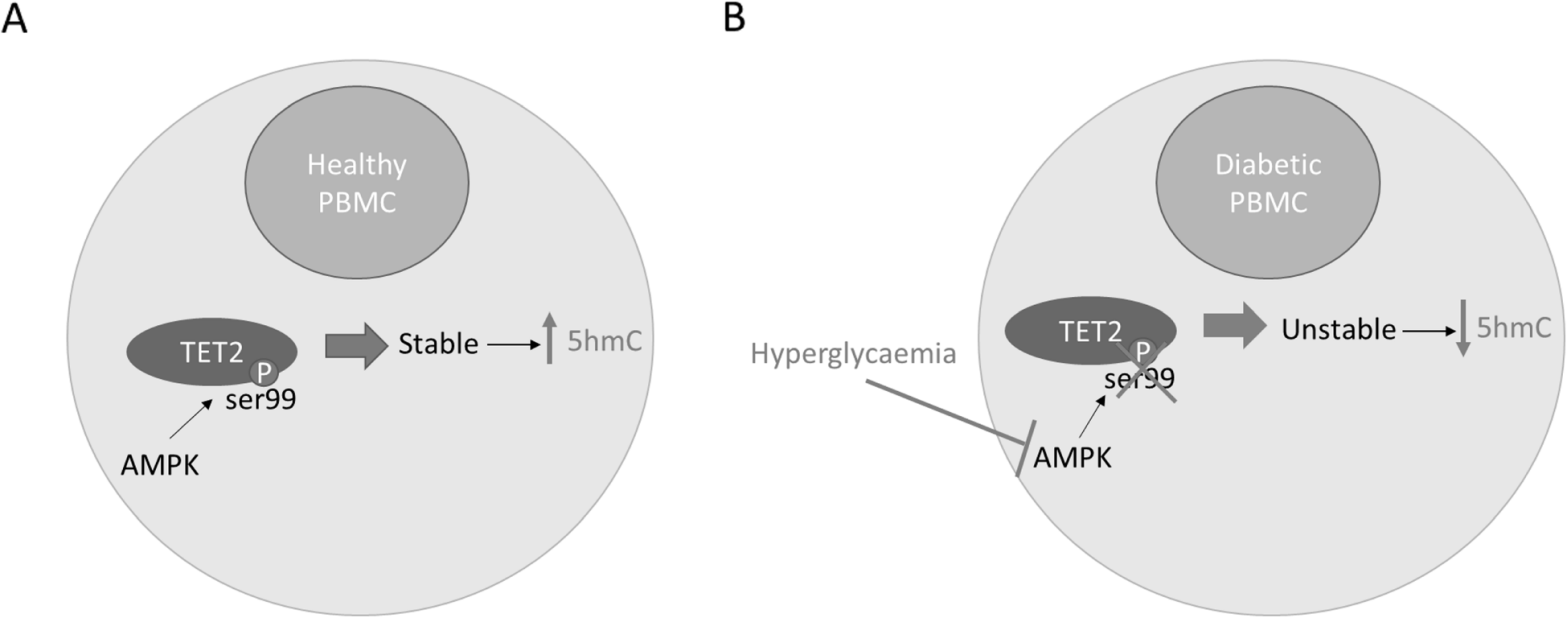
Hyperglycaemia impairs adenosine monophosphate-activated protein kinase (AMPK)-mediated stabilisation of ten-eleven translocation (TET)2. **a** In peripheral blood mononuclear cells (PBMCs) from healthy controls, AMPK phosphorylates TET2 at serine 99, thereby stabilising it and increasing levels of its product, 5-hydroxymethylcytosine (5hmC). **b** Hyperglycaemia impairs AMPK mediated phosphorylation of TET2 in PBMCs from diabetic patients, leading to destabilisation and reduced activity of TET2

Post-translational modifications of 2-OGDD demethylases have not thus far been extensively studied. However, in a significant recent study, Wu et al. found that TET2 is phosphorylated at serine 99 by 5'AMP activated protein kinase (AMPK) in peripheral blood mononuclear cells (PBMCs) (Fig. 6) [138]. High glucose levels disrupted AMPK-mediated phosphorylation, leading to decreased stability of the TET2 protein (Fig. 6) [138]. This is likely to account for their finding that global levels of 5hmC are lower in PBMCs from type 2 diabetic patients compared to healthy donors (Fig. 6) [138]. This is perhaps the strongest evidence, to date, of high glucose levels affecting the activity of a 2-OGDD, and it would be interesting to investigate whether this finding extends to other cell types including ECs, and whether other 2-OGDDs including JmjCs are similarly affected.

Several JmjC family members have been reported to possess phosphorylated peptides [139] including lysine demethylase-3A and -7C (KDM3A and KDM7C) which have been shown to be specifically phosphorylated by mitogen- and stress-activated protein kinase 1 (PK1) and PKA respectively [140, 141]. In the latter case, KDM7C is reportedly inactive unless phosphorylated by PKA, which enables it to associate with the DNA binding protein, AT-Rich Interaction Domain 5B (ARID5B) and demethylate H3K9me2 [141]. This highlights the potential for phosphorylation to activate 2OGDDs either directly or by promoting the formation of active complexes. Investigating whether hyperglycaemia affects the phosphorylation of 2-OGDD members other than TET2, and the effects of this on endothelial cell function, could be a valuable area of study.

## Conclusions and future perspectives

The challenge to develop better preventative treatments to alleviate the escalating problem of CVD associated with T2D necessitates a detailed mechanistic understanding of the disease aetiology. The causal links between hyperglycaemia, altered epigenetic patterns and endothelial dysfunction now appear irrefutable. It is therefore now critical to understand how epigenetic patterns are normally regulated in the endothelium and how hyperglycaemia might dysregulate this. In addition, such a biochemical understanding of the relationship between high glucose exposure and epigenetic regulation would serve as powerful proof of the concept of the epigenetic basis of disease.

It is noteworthy that ED may, in part, be initiated or exacerbated by changes in other cell types, such as leukocytes participating in the inflammatory response which is strongly associated with cardiovascular disease [142]. In addition, dysfunctional vascular smooth muscle cells can contribute to the CVD associated with diabetes [143]. Therefore, although the focus here has been ECs, hyperglycaemia-mediated epigenetic changes in immune cells and other vascular cells are also highly relevant. Further studies on these cell populations are important to understand not only generalised mechanisms of 2OGDD regulation but also the involvement of these other cell types in the development of CVD in T2D.

The fact that many epigenetic modifiers use energy metabolites has initiated a large field of study aimed at elucidating the link between epigenetic adaption to the environment and the progression of non-communicable diseases, most notably cancer [39, 144, 145]. The clear associations between altered epigenetic methylation patterns and diabetes-associated CVD suggest that the dysregulation of the methylome is of critical importance in its aetiology [34–38]. In addition, altered methylation patterns are capable of inducing lasting gene expression changes, which could contribute to developmental priming in offspring of diabetic mothers, which perpetuates the disease burden throughout successive generations [5, 6]. Therefore, understanding the epigenetic mechanisms at play could have a far-reaching impact.

The substrate and co-factor requirements of the 2-OGDD family of epigenetic demethylases suggest they may act as sensors and integrators of not only energy metabolic fluxes (for instance, glycolysis versus OxPhos) but also O_2_ availability and cellular redox. Their activities may further be impacted by post-translational modifications, perhaps also modulated by hyperglycaemia. However, the relationship between glucose exposure, the bioavailability of the obligate substrates/cofactors in endothelial cells and the catalytic activities of these enzymes remains poorly understood and the available data is sometimes contradictory. Investigating this may aid our understanding of hyperglycaemia-induced ED, and potentially inform significant improvements in the future prevention and management of CVD in diabetes.

Intriguingly, somatic mutations in 2-OGDD enzymes have increasingly been found to associate with other pathologies, most notably certain cancers. Thus, mutations in TET enzymes, and in particular TET2, have been identified in haematological cancers including myelodysplastic syndromes [146], myeloproliferative neoplasms [147], mixed lineage leukaemia [148], T cell lymphomas [149] and B cell non-Hodgkin lymphoma [150]. A recent study also revealed that TET2 is one of the most commonly mutated genes in peripheral blood cells of individuals with clonal haematopoiesis (benign clonal expansion of blood cells), which associated with a higher incidence of atherosclerosis (assessed as coronary artery calcification), myocardial infarction and coronary heart disease [151]. Thus, (dysregulated) TETs are associated with haematological and (non-diabetes-associated) cardiovascular diseases [146–151]. Mutations in JmjCs have also been associated with cancers, developmental and inflammatory diseases, which have been reviewed elsewhere [152]. The study of the potential epigenetic basis of these pathologies is therefore a field of active investigation.

It is imperative also to gain a full understanding of the epigenetic mechanisms underlying the cell-type-specific transcriptional regulation of the genome. The recent rise in single-cell epigenomic analyses, coupled with transcriptomics, shows great potential in understanding subtle molecular differences in heterogeneous cell populations [153]. Further, the development of CRISPR-Cas technologies facilitating RNA-guided epigenetic regulators to specifically edit the epigenetic code will further elucidate the functional consequences of specific epigenetic marks [154]. The application of these technologies to ECs challenged with stressors, such as high glucose, could provide unprecedented insight into the epigenetic basis of gene regulation and endothelial (dys)function.

Given that modulating DNA methylation status or histone post-translational modifications can co-ordinate transcriptional silencing or activation of many genes, the regulators involved in this process, including TETs and JmjCs, may be attractive targets for epigenetic-based therapies. These could potentially co-ordinate the broad transcriptional changes that would be required to maintain or restore normal cell function in unfavourable conditions (such as hyperglycaemia). Epigenetic modifiers, such as inhibitors of HDACs and DNA methylation, are already undergoing clinical trials for some cancers, and a number of these have gained approval from the Food and Drug Administration (reviewed in [155, 156]). It is likely that the number of epigenetic modifying drugs available and the number of conditions for which they are used will expand, informed by further research.

## Data Availability

Not applicable; no new data are presented.

## Abbreviations

CVD: Cardiovascular disease
ED: Endothelial dysfunction
TET: Ten-eleven translocation
JmjC: JumonjiC
T2D: Type 2 diabetes
EC: Endothelial cell
HUVEC: Human umbilical vein endothelial cell
AGE: Advanced glycosylation end-products
HAEC: Human aortic endothelial cell
SAM: S-adenosyl methionine
2-OGDD: 2-oxoglutarate-dependent dioxygenase
2-OG: α-ketoglutarate
DNMT: DNA methyltransferase
5mC: 5-methylcytosine
5hmC: 5-hydroxymethylcytosine
5fC: 5-formylcytosine
5caC: 5-carboxylcytosine
TDG: Thymine DNA glycosylase
HAT: Histone acetyltransferase
HDAC: Histone deacetylase
HMT: Histone methyltransferase
LSD: Lys-specific demethylase
HIF: Hypoxia-inducible factor
PHD: Prolyl hydroxylase
OxPhos: Oxidative phosphorylation
TCA: Tricarboxylic acid
BAEC: Bovine aortic endothelial cells
LDH: Lactate dehydrogenase
MDH: Malate dehydrogenase
2HG: 2-hydroxyglutarate
ROS: Reactive oxygen species
MMP-9: Matrix metalloproteinase-9
SOD: Superoxide dismutase
PRC2: Polycomb repressive complex 2
OGT: O-GlcNAc transferase
HCF1: Host cell factor 1
PBMC: Peripheral blood mononuclear cell
KDM: Lysine demethylase
ARID5B: AT-rich interaction domain 5B
AMPK: 5'AMP activated protein kinase
PARP: Poly(ADP-ribose) polymerase
